# Cartographic Infrastructures: Geographical Pathology, Tumour Safaris, and Colonial Networks in British East Africa

**DOI:** 10.1177/03063127251364513

**Published:** 2025-08-25

**Authors:** David Reubi, Thandeka Cochrane

**Affiliations:** King’s College London, London, UK

**Keywords:** geographical pathology, mapping, colonial medicine, epistemic infrastructure, Burkitt’s lymphoma

## Abstract

In this article, we explore infrastructures—human, epistemic, and material—that enable the creation of maps and the stories they tell about the world. We develop the concept of ‘cartographic infrastructures’ to reveal the hidden and fragile political, scientific, and social worlds that undergird the production of maps and the truths they bring into being. To illustrate this, we examine a series of highly influential maps published in the early 1960s by Denis Burkitt—a colonial doctor working in British East Africa—showing the geographical distribution of a pediatric tumour in Africa. By correlating the tumour with altitude and rainfall, the maps suggested it had a viral origin, a finding that profoundly affected cancer research and helped accelerate the development of viral oncology. Using reports, diaries, and photos left behind by Burkitt and others, we excavate the cartographic infrastructures that underpinned his maps and viral hypothesis. First, we examine how the research tradition of geographical pathology—with its focus on environmental factors in carcinogenesis and its interest in Africa as a laboratory for cancer research—provided Burkitt with the theories, networks, and funding necessary to conduct his work. Second, we examine the surveillance practices, ranging from tumour safaris to geographical plotting, that enabled Burkitt to generate and interpret the data underlying his maps. Third, we analyze the communities of white colonial medical officers and missionary doctors spread across Africa with their shared imaginaries of race, empire, and adventure on which Burkitt relied to conduct his surveys.

There is a rich body of scholarship on disease mapping in the humanities and social sciences. Much of this scholarship has focused on the late 18th and 19th centuries, a period when mapping came to play a central role in medicine and public health. The maps studied range from medico-topographical surveys used by European military doctors during the colonization of Asia and Africa to sanitary maps depicting poverty and insalubrity, deployed by health officials fighting epidemics in New York, Hamburg, and Bombay (e.g., Arnold, 1993; Gilbert, 2004; Harrison, 2000; Moussy, 2015; Schulten, 2012). More recently, scholars have also examined disease mapping in the 20th and 21st centuries. While some have studied how colonial medical officers employed cartographic methods to control tropical diseases like malaria, others have analyzed global epidemiological surveillance initiatives charting AIDS and other contemporary epidemics (e.g., Barney, 2014; Cummiskey, 2020; Engelmann, 2020; Kearns, 2017; Oliver, 2006).

For most of these scholars, the central concern is the power of disease maps to shape the world, a concern that has roots in both science and technology studies and critical geography. In science and technology studies, maps are often viewed as scientific tools akin to diagrams and charts, which scientists use to ‘argue, prove’, and ‘convince’ others to ‘take up a statement’ and ‘make it more of a fact’ (Latour, 1990, pp. 23–24). In critical geography, maps are frequently conceived of as ‘rhetorical texts’ with ‘ideological contours’ that ‘operate on social reality’ to reinforce the dominant ‘cultural models’ (Harley, 2001, pp. 159–163). Drawing on these ideas, these scholars have shown how disease maps legitimized 19th-century assumptions about the insalubrious nature of the tropics, validated mosquito-transmission models in 20th-century malaria research, and shaped contemporary narratives about a global obesity epidemic (e.g., Cummiskey, 2020; Harrison, 2000; Oliver, 2006). Some scholars have also paid close attention to the political mobilization of medical maps, documenting how they have been used for purposes ranging from exploiting colonial territories and governing the Victorian poor to advocating for research funding and creating new markets for pharmaceuticals (e.g., Barney, 2014; Gilbert, 2004; Moussy, 2015).

While these scholars have richly articulated what medical maps do, they have had less to say about the infrastructures that make medical maps possible. When they do look at the production of maps, they tend to focus on the wider political and intellectual context in which maps are made. For example, historians studying Victorian sanitary maps have located these within the public health efforts, enthusiasm for vital statistics, and interest in the association between climate, topography, and disease that characterize this era (Arnold-Forster, 2020; Gilbert, 2004; Schulten, 2012). Similarly, scholars analyzing medico-topographical surveys have shown how these were linked to European imperial agendas and the military conquest of South Asia and the Maghreb (Arnold, 1993; Moussy, 2015). Few scholars go beyond this contextualization work to give a granular description of the medical theories, scientific networks, surveillance infrastructures, and funding opportunities that enable the making of disease maps.

In this article, we examine the epistemic, material, and human infrastructures that make the production of cartographic knowledge possible. For this we develop the concept of ‘cartographic infrastructures’ to shed light on the rationalities, practices, and socialities that enable the creation of disease maps and the truths they bring into being. Maps tend to obscure the infrastructures that undergird their formulation and existence. We suggest that attending to cartographic infrastructures helps us to look behind maps and untangle the multiplicities of worlds that enable them. Moreover, by stressing these infrastructures’ contingent and ephemeral nature, we remind readers that, for all their world-making powers, maps and the knowledge they carry with them are inherently fraught and fragile.

To illustrate the analytical power of this concept, we examine a set of maps charting the geographical distribution of Burkitt’s lymphoma, a jaw tumour affecting children, across sub-Saharan Africa (see Figures 1 and 2). Highly influential and celebrated within and beyond the medical world, these maps were published in leading medical journals in the early 1960s—during the era of African decolonization—by Denis Burkitt, a doctor working for the British colonial administration in Uganda (Mika, 2022). The maps showed how the distribution of the jaw tumour across Africa was dependent on altitude and rainfall, suggesting that the tumour might be caused by a virus transmitted by a mosquito. By giving credence to the then still-peripheral idea that cancer could be caused by viruses, these maps had a profound impact on international cancer research and helped accelerate the development of viral oncology—a field that, today, has grown into a major biomedical research enterprise (Scheffler, 2019). These maps have remained a reference for researchers working on cancer in Africa and continue to feature in medical hagiographies on Burkitt (e.g., Cummings, 2022). As a critical event in the development of viral oncology, they have also found a place in histories of medical mapping, appearing in numerous works and anthologies on the topic (e.g., Koch, 2017).

**Figure 1.**
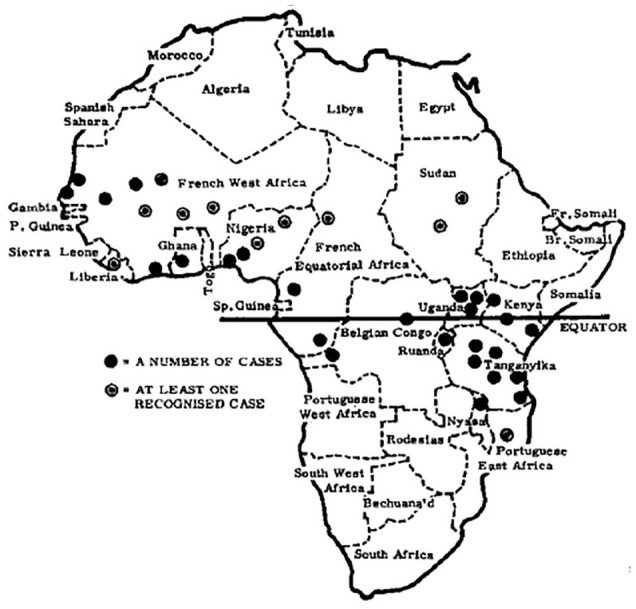
Map showing the distribution of Burkitt’s lymphoma in Burkitt and O’Conor’s 1961 article in *Cancer*.

**Figure 2.**
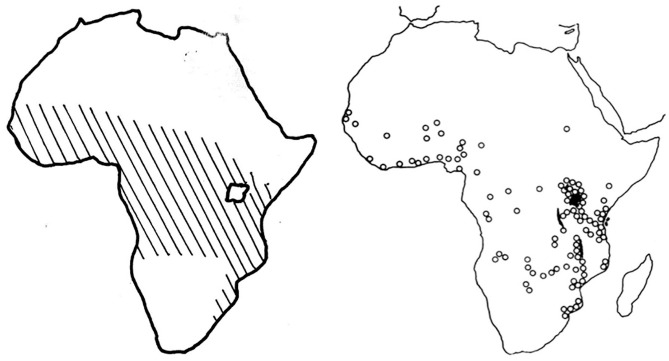
Maps showing the ‘lymphoma belt’ across Africa as they appeared in Burkitt’s 1961 article in the *East African Medical Journal* (left) and in his 1962 article in the *British Journal of Cancer* (right).

Burkitt et al. left behind substantial archival documents, from personal diaries and correspondence to reports and photos that allow us to explore the cartographic infrastructures underlying his maps. We start by examining the research tradition and networks of geographical pathology that enabled Burkitt’s work on lymphoma. From there, we explore the surveillance and cartographic techniques undergirding Burkitt’s maps. Lastly, we examine the colonial medical communities that helped Burkitt carry out his research on lymphoma. But before doing so, we discuss the concept of cartographic infrastructure and introduce Burkitt’s maps in more detail.

## Cartographic infrastructures

The word infrastructure has a long genealogy (Carse, 2017). Coined by 19th century railway engineers, its usage was long restricted to transport projects. Specifically, it was used to distinguish the construction work sitting beneath unlaid tracks (roadbeds) from the superstructure of rails and train stations situated above the tracks. The term suggested ‘the integration of parts’ to a whole with an added notion of ‘depth or hierarchy’ (Carse, 2017, p. 30). The use of the term expanded after World War II, when military experts started using it to refer to the fixed installations necessary for the deployment of modern armed forces, such as airfields and radar stations. At the same time, development specialists began arguing that large-scale infrastructure projects such as roads and electrical networks were critical for economic growth, and later expanded the term to encompass more ‘intangible aspects like health, social attitudes, [and] industrial skills’ (Carse, 2017, p. 34). It is this layered notion of infrastructure that science and technology scholars have mobilized to interrogate the large technological systems—such as power grids, information systems, and transportation networks—that characterize modern societies. For these scholars, the term infrastructure is a call to ‘unearth’ and analyze the ‘system of substrates’ and ‘hidden mechanisms subtending’ these sorts of ‘large-scale technical systems’ (Star, 1999, pp. 377, 380).

The influential work of Star and Bowker offers a good illustration of this notion of infrastructure as an analytic lens. For them, infrastructure is a ‘mixture of physical entities’, ‘conventional arrangements’ and ‘communit[ies] of practice’ that do ‘the real work … of knowledge production’ (Bowker & Star, 2000, pp. 34–35, 39). Infrastructure is an assemblage that weaves together ‘profoundly mundane’ elements, from ‘paper forms [and] plugs’ to ‘classification systems’, and ‘organizational routines’ to ‘people whose work goes unnoticed’ (Bowker & Star, 2000, p. 39; Star, 1999, pp. 377, 386–387). Bowker and Star (2000, p. 34) suggest that infrastructure is typically invisible, that it has a ‘tendency to disappear’, to ‘fade into the woodwork’. It is ‘the forgotten, the background, the frozen in place’, which only ‘becomes visible when it breaks: The server is down, the bridge washes out, there is a power blackout’ (Star, 1999, pp. 379, 382). Infrastructure, they add, is also ‘fragile’ (Star, 1999, p. 387). Its assemblage and operation depend on ‘local and situated contingencies’, and its lifespan is inevitably limited due to the ‘decay and degradation’ that affect all materials (Gupta, 2018, p. 76; Star, 1999, p. 387). For science and technology studies scholars, the task is to perform what Bowker and Star (1996) call an ‘infrastructural inversion’, to take the ‘behind-the-scenes, boring, background processes’ of knowledge production and ‘bring their contribution to the foreground’.

Building on this work, we see cartographic infrastructures as assemblages of logics, materialities, and socialities that undergird disease maps and the stories they tell about the world. Although the elements that make up these assemblages are closely entangled, for heuristic reasons we distinguish between three types of infrastructural components. First, there are the research traditions and networks that drive mapping efforts and shape both the maps and the truths they encapsulate. These ‘epistemic infrastructures’, as Murphy (2017, p. 6) calls them, do not just include experimental settings and theories about disease aetiology, but also more mundane arrangements like collaborative partnerships, spaces for scientific exchange, funding flows, and research facilities. Second, there are the techniques that enable the generation, presentation, and interpretation of data upon which maps are based. These surveillance infrastructures, as we call them, encompass both methodological practices—such as social surveys and epidemiological modelling—and material components, including paper tools and road networks, upon which these methodologies depend. Third, there are human infrastructures or what Simone (2004, p. 407) calls ‘people as infrastructure’: communities of practices and networks whose shared political horizons, social practices, and professional values help shape medical maps. All these infrastructures are typically erased in maps and the medical realities they help bring into being. The notion of cartographic infrastructure helps us to unearth and illuminate these hidden mechanisms behind maps, revealing the often fraught dynamics of knowledge production. Furthermore, by documenting the lives of these mechanisms, from their contingent assemblage to their slow decay, this approach draws our attention to the impermanent and fragile nature of cartographic infrastructures.

The epistemic, material, and human substrates revealed through an infrastructural approach are critical in shaping the stories that maps tell about the world and are of interest to scholars focused on cartographic power. At the same time, scholars who work on cartographic infrastructures want to keep in mind that these infrastructures have themselves been informed and shaped by past maps and mapping practices. Thus, in a recursive and layered epistemic process, current map-making practices are dependent upon and informed by earlier maps and the medical and political worlds they bring into being. Later in this article, we draw attention to an element of this recursive epistemic dynamic by elucidating how previous colonial medical maps and mapping techniques form a central part of the surveillance infrastructure underlying Burkitt’s lymphoma charts.

## Denis Burkitt and the African lymphoma maps

The maps we examine here to illustrate the analytic power of cartographic infrastructures were published by Denis Burkitt in the early 1960s across a range of medical journals, including *Nature, The British Medical Journal (BMJ), Cancer*, and *The East African Medical Journal (EAMJ)*. All the maps featured the African continent with dots or cross-hatchings marking the locations where the child jaw lymphoma had been recorded (see Figures 1 and 2). As Burkitt noted, the ‘area of tumour distribution’ shown in the maps constituted a ‘lymphoma belt’ stretching ‘across tropical Africa with a tail running down the East coast’ (Burkitt, 1961a, p. 511, 1962a, p. 379; Burkitt & O’Conor, 1961, p. 268). He argued that the geographical distribution of the lymphoma suggested an association with ‘external environmental factors’ rather than ‘racial or tribal factors’, as some had proposed (Burkitt & O’Conor, 1961, pp. 261, 267). Specifically, Burkitt observed that the tumour belt was ‘altitude’ and ‘humidity dependent’, leading him to speculate that ‘some virus’ transmitted by ‘a mosquito’ might be the ‘responsible agent’ for the tumour (Burkitt, 1962b, p. 234, 1962d, pp. 1019, 1022).

Trained at Trinity College Dublin, Burkitt had joined the British Colonial Medical Service after World War II and been posted to East Africa. By the time he first encountered the African lymphoma in the late 1950s, he was working as a surgeon for the colonial administration at Mulago Hospital in Kampala. Burkitt describes his incredulity when first seeing a young African child disfigured by the extensive swelling in all four quadrants of the jaw that is characteristic of the tumour. Faced with ‘this queer, unidentified thing’ so different from anything he had seen in the UK, Burkitt’s curiosity was sparked, and he spent the next ten years trying to understand the affliction (Burkitt, cited in Glemser, 1970, pp. 49–50). His early work on the topic was mainly concerned with identifying the tumour as a singular disease and describing its clinical features, histology, and treatment. It was only later that Burkitt became interested in the tumour’s geographical distribution and the clues this could provide about its aetiology. To establish the tumour’s geographical distribution, he first carried out a postal survey of doctors from across the continent, from British and Portuguese East Africa to French West Africa. Then, in late 1961, he carried out, with two friends, what he called a ‘tumour safari’: He drove 16,000 kilometres over ten weeks to interview doctors at hospitals across eight countries from Uganda to South Africa (Burkitt, 1962c). In early 1962, he carried out two additional tumour safaris: one in the Belgian colony of Ruanda-Urundi (present-day Rwanda and Burundi) and another to the newly independent nations of Ghana, Nigeria, and Congo. His lymphoma maps were the products of these survey-safaris.

Burkitt’s maps and the association they suggested between cancer and viruses had a profound influence on international cancer research. They provided critical support for what was still a peripheral idea—that viruses could be carcinogenic—thereby significantly accelerating the development of the nascent field of viral oncology (Aviles, 2024; Cummiskey, 2024; Morgan, 2022; Scheffler, 2019). In 1964, Anthony Epstein and his team at Middlesex Hospital in London were able to confirm the link between cancer and viruses suggested by Burkitt’s maps. Indeed, directly inspired by Burkitt’s work and in collaboration with him, Epstein and his team identified a virus—later called the Epstein-Barr virus (EBV)—in cells taken from a child with the lymphoma at Mulago (Epstein et al., 1964). All this generated much excitement and ample funding opportunities from research agencies such as the U.S. National Cancer Institute (NCI), helping the development of what is now known as viral oncology (Aviles, 2024; Scheffler, 2019). The maps also transformed Burkitt’s life, turning him into a star in the medical world and beyond. In 1963, delegates at the Annual Conference of the International Union against Cancer (UICC) in Paris named the lymphoma after him. Burkitt was invited on extensive, months-long speaking tours in America, Europe, and Asia. He and his maps were featured in the media, from local papers in British Africa to major American titles, and in a book titled *Mr Burkitt and Africa* (Glemser, 1970).

## Epistemic infrastructures: Geographical pathology in colonial Africa

In the following sections, we examine some of the major infrastructures that undergird Burkitt’s influential lymphoma maps. We begin by examining a key epistemic infrastructure that made Burkitt’s maps and his hypothesis about viral oncogenesis possible—the assemblages of scientific theories, expert networks, funding arrangements, and research facilities associated with geographical pathology in colonial Africa. With a history going back to the 19th century, geographical pathology was a research tradition that became influential in international cancer research after World War II (Cochrane & Reubi, 2023; Mueller, 2019). Championed by some of the biggest names in the field, from Pierre Denoix to Richard Doll, it sought to advance the knowledge of the causes of cancer by comparing populations living in different locations and exposed to varying nutritional, social, economic, and other environmental factors. A pivotal moment in the development of this research tradition was the Conference on Geographical Pathology and Demography of Cancer, held in Oxford in 1950, which brought together researchers from Europe, North America, South Africa, and India. Geographical pathology, according to the conference participants, was the study of ‘geographical variations in the incidence and behaviour of cancer’ to shed light on ‘the part played by race, nutrition, infections, and environment … in carcinogenesis’ (Clemmesen, 1951, pp. 9–10). The ‘ultimate aim’ of this type of research, they claimed, was ‘the prevention of cancer in man’ (Clemmesen, 1951, p. 628). Indeed, they believed that by identifying the social, economic, and environmental factors that caused cancer, studies in geographical pathology enabled people to alter their environments and ways of living to avoid exposure to these dangers.

While proponents of geographical pathology were interested in studying cancer ‘in all parts of the world’, they thought that ‘observations from less sophisticated’ parts of the planet could be especially ‘fundamental to [advancing their] ideas on the aetiology of human cancer’ (Clemmesen, 1951, pp. 12–13). Africa and Africans, in particular, were of great interest. This was already evident at the 1950 Oxford conference and was reiterated at a follow-up Symposium on ‘Cancer in the African Negro’ held in Leopoldville in the Belgian Congo in 1956. Echoing early 20th-century notions of ‘Africa as a living laboratory’ that was critical in producing Western medical knowledge (Tilley, 2011), researchers at the symposium believed that by relating ‘different types of neoplastic disease to environmental factors such as nutrition, parasitic and other infectious diseases’, ‘studies on cancer in Africa south of the Sahara’ offered a great ‘opportunity’ to ‘contribute to medical science’ (Clemmesen, 1957, p. 2). The participants especially recommended that studies be done on ‘types of neoplastic disease occurring with frequencies different from those expected [in the West]’ (Clemmesen, 1957, p. 3). Similarly, writing in *The Lancet* in the early 1950s, Jack Davies, a pathologist at Makerere University and a leading voice in African cancer research, called for more attention to ‘be paid to the problems of cancer in Africa’ (Croot & Davies, 1952, p. 158). He pointed out that the ‘cancer picture’ in Africa was ‘widely different’ from that ‘seen in Europe’, with a ‘gross excess’ of cancers that were ‘extreme rarities in Britain’, such as ‘primary liver cancers’ (Croot & Davies, 1952, p. 158). As he explained in later writings, it was ‘these differences’, in terms of both ‘cancer patterns’ and possible ‘environmental causes’, such as ‘soils, climates, diets, [and] tribal customs’, that made ‘Africa such a valuable field for studies in the aetiology of cancer’ (Davies, 1963, p. 534). These ideas of African bodies and environments as different were, of course, not new but built on models of human progress that have long haunted Western imaginaries and construed Africa as unsophisticated, primitive, and perpetually behind Euro-America (Cochrane & Reubi, 2023).

The epistemic assemblage that constituted geographical pathology was not limited to scientific theories about cancer in Africa; it also included more prosaic elements, such as collaborative arrangements, scientific forums, research facilities, and funding flows. Following the Oxford conference, the UICC set up a Committee on Geographical Pathology. Led by Harold Stewart from the NCI, the Committee built an international network of researchers working in the field and supported their research. Given the prominence of the network’s research in Africa, an African Sub-Committee was created at the 1956 Symposium in Leopoldville. The Sub-Committee brought together and coordinated the work of mostly European cancer researchers based in Africa, such as Jack Davies and Hugh Trowell in British East Africa, George Oettlé and John Higginson in South Africa, Robert Camain in French West Africa, and Manuel Prates in Portuguese East Africa. Their work was made possible by multiple funding opportunities for research on cancer, especially those from British sources, such as the British Empire Cancer Campaign and the UK Medical Research Council (MRC) (Cochrane & Reubi, 2023).

More generally, the surge in geographical pathology research on cancer in Africa was enabled by renewed efforts of European imperial powers to develop their colonial possessions on the continent following World War II. Indeed, despite facing increasing anti-imperial mobilization, powers like Britain and France saw their colonies in Africa as critical to their post-war economic recovery and were determined to keep and consolidate them (Cooper, 2002). Framed within postwar development discourses, initiatives like the British Colonial Development and Welfare Act led to increased investments in the colonies, from the extension of transport networks to the building of new schools. Science was central to this post-war ‘developmental colonialism’, with state budgets for scientific research in the colonies markedly increasing (Cooper, 2002, p. 37). Medical research was no exception, with the creation of new universities, field stations, and hospitals, as well as the establishment of new funding bodies such as the British Colonial Medical Research Committee, formed by the Colonial Office together with the MRC (Reynolds & Tansey, 2001).

**Figure 3.**
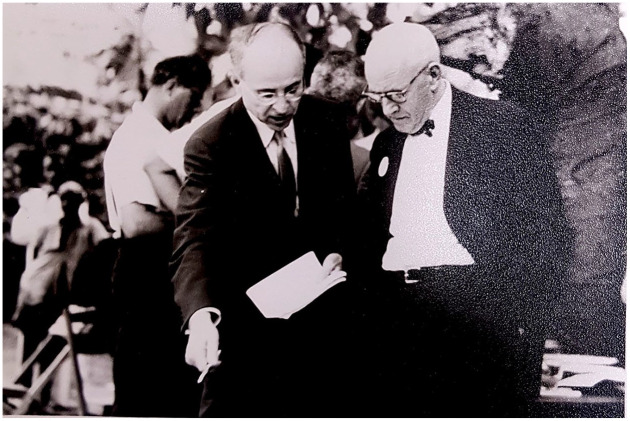
Harold Stewart (right), Secretary of the UICC Committee on Geographical Pathology, speaking with a colleague at the 1956 UICC Symposium on Liver Cancer in Kampala. Source: U.S. National Library of Medicine (Harold Stewart Papers, Box 86, Folder 18).

Kampala, home to Makerere University and Mulago Hospital, where Burkitt worked, was one of the epicentres of geographical pathology and cancer research in Africa. Indeed, alongside colleagues such as Trowell, Davies had helped transform Kampala into a flourishing international research hub that led postwar cancer studies in Africa (Mika, 2022). With funding from the British Empire Cancer Campaign, he had established Africa’s first population-based cancer registry, set up a Cancer Research Committee, conducted some of the first surveys on cancer in the region, and published in leading medical journals (Davies et al., 1958, 1962). Davies and his colleagues often presented their work at Mulago’s Staff Clinical Meetings. For example, starting in the late 1940s, Davies regularly outlined the frequencies and types of cancer in Kampala (e.g., EAMJ, 1949, 1956). Importantly, in 1956, two of Davies’ collaborators, Pritam Singh and J. Cook, presented a pathological-clinical study of 78 ‘tumours of the jaw’ among African children and adults that they had come across while working at Mulago (EAMJ, 1956). They noted that the ‘figures from the Kampala Cancer Registry’ confirmed that ‘jaw tumours occur more frequently in Uganda than in many other parts of the world’ (Singh & Cook, 1956, p. 383). They also remarked that one type of tumour ‘particularly occur[red] in children’ and was ‘striking’ in terms of its ‘size’ and the ‘grotesque deformities’ it generated (Singh & Cook, 1956, p. 387). Davies and Trowell were also active members of the UICC African Sub-Committee on Geographical Pathology, hosting the Sub-Committee’s Symposium on Liver Cancer at Makerere in 1956 (Clemmesen, 1957; see Figure 3).

While not part of the UICC Committee on Geographical Pathology, Burkitt actively engaged with the world of geographical pathology, interacting with many of the experts and funders studying malignancy in Africa and attending their conferences and workshops. As a surgeon at Mulago from 1948 to 1964, Burkitt knew most researchers working on geographical pathology and cancer in Kampala. Trowell, for example, was a close friend whom he had met on the train from Nairobi to Mombasa back in 1947. They regularly lunched together at Mulago, often met with their families on the weekend, and, after their return to the United Kingdom, collaborated on research about the relationship between diet and oesophageal cancer and published their influential book *Western Diseases* in 1981 (Cummings, 2022). Burkitt also knew Davies and was familiar with his work from attending Mulago’s Staff Clinical Meetings. While they published on the lymphoma together, the two men had a fraught relationship marked by intense professional rivalry (Mika, 2022). Kampala, as an international hub for geographical pathology, also allowed Burkitt to meet other key figures in the field. For example, Burkitt was in Kampala in 1956, when some of the most influential researchers in geographical pathology, like Stewart, Denoix, Camain, and Prates, visited for the UICC Symposium on Liver Cancer. Similarly, in 1957, Burkitt got to meet Oettlé from the African Sub-Committee for Geographical Pathology (Glemser, 1970, p. 69). During their conversation, Oettlé told Burkitt that the African lymphoma did not occur in South Africa, leading Burkitt to examine the tumour’s geographical distribution. The two men, who shared a strong Christian faith, remained close friends, with Burkitt visiting him in Johannesburg on his 1961 tumour safari (Cummings, 2022). That same year, Burkitt also met Harold Himsworth, the head of the MRC and a major financial backer of geographical pathology, at a ‘Symposium on Cancer’ hosted by Davies at Makerere University (Cummings, 2022).

It was through his engagement with the epistemic world of geographical pathology that Burkitt acquired the intellectual, material, and financial tools to work on the African lymphoma. To start, it was by attending geographical pathology forums and interacting with experts in the field that Burkitt was made aware of the lymphoma itself. Specifically, it was Davies’ collaborators, Singh and Cook, who introduced Burkitt to the lymphoma during their 1956 presentation at Mulago’s Staff Clinical Meeting (EAMJ, 1956, p. 71). A year later, it was Trowell who showed Burkitt his first patient with the tumour at Mulago (Glemser, 1970). More broadly, it was by participating in these forums and talking to these experts that Burkitt became convinced that Africa, with its different ‘cancer patterns’ and ‘environmental factors’, offered ‘exciting possibilities’ for cancer research (Burkitt, 1969, pp. 82–84; Hutt & Burkitt, 1965, p. 719). Crucially, it was also through these forums and experts that Burkitt was introduced to the ideas of geographical pathology, providing him with the intellectual framework for his work on the lymphoma. Burkitt clearly saw his work as a ‘geographical study’ (Hutt & Burkitt, 1965, p. 720). This, he explained, meant studying ‘variations in disease patterns’ in ‘different parts of the world’ to ‘provide clues to the aetiology of these conditions’, as he had done with the lymphoma (Burkitt, 1963a, p. 1; Burkitt et al., 1963, p. 6). As Burkitt (1963a, p. 1, 1963b, p. 2) recognized, his ‘geographical approach to cancer problems’ was built on the ‘excellent work that [had] been accomplished in Africa in the field of geographical pathology’. He especially acknowledged his debt to Davies’s ‘pioneer work’, adding that it was ‘Davies who first suggested to [him] that [the jaw] tumour might be virus-induced’ (Burkitt, 1961a, p. 512; Hutt & Burkitt, 1965, pp. 719, 722). Lastly, the networks of geographical pathology also gave Burkitt access to funding, with him securing financial support for his tumour safaris from the MRC after meeting with Himsworth at a cancer symposium in Kampala (Burkitt, 1962c, p. 386).

## Surveillance infrastructures I: The medical safari-as-survey

The epistemic infrastructure of geographical pathology, although important, was not the only condition of possibility for Burkitt’s maps. Also critical were the surveillance infrastructures and practices that Burkitt used to produce the data for his maps. Geographical pathologists like Davies, Higginson, and others had already begun experimenting with new surveillance techniques to study cancer in Africa in the postwar period, such as setting up cancer registries and running social surveys in cities like Kampala, Johannesburg, and Lourenço Marques (Davies et al., 1958; Oettlé & Higginson, 1956; Prates & Torres, 1965). While Burkitt was likely familiar with these methods, the surveillance techniques he devised and employed in his lymphoma research were quite different and relatively unique in cancer research.

Burkitt called his approach to surveillance ‘the safari approach’, ‘tumour safaris’, or ‘geographical pathology safaris’ (Burkitt, 1962d, p. 1019; Burkitt et al., 1970, p. 197). As he noted, the word safari means ‘journey’ or ‘travel’ in Kiswahili. Although Burkitt used the term in this more technical sense, it also carries connotations of adventure and game hunting—associations that were not entirely absent from his method. Burkitt described a ‘safari’ as a ‘method of research’ that ‘depends upon travel’ and ‘has as its basis personal visitation’ to hospitals across a vast territory to consult with local doctors and review operation registers to determine the geographical distribution of a particular cancer (Burkitt et al., 1970, p. 197). Or as he put it elsewhere, ‘safaris’ were ‘journeys’ that were done ‘to provide “geographical biopsies”—that is, detailed surveys of portions of Africa containing “tumour-bearing” and “tumour-free” areas’ (Burkitt et al., 1970, p. 197). As Burkitt (1964a) acknowledged, his safari-as-survey methodology stemmed from a key practice in the work of colonial medical officers—the medical safari. Generally based in the main urban centre of a particular district where they oversaw the local hospital, these officers were required to go on ‘regular medical safaris to oversee the running of rural dispensaries [in their district] and provide help for patients needing specialist treatment’ (Crozier, 2007, p. 91). As a colonial medical officer for about 15 years, Burkitt was well-versed in medical safaris, having conducted countless such trips himself.

**Figure 4.**
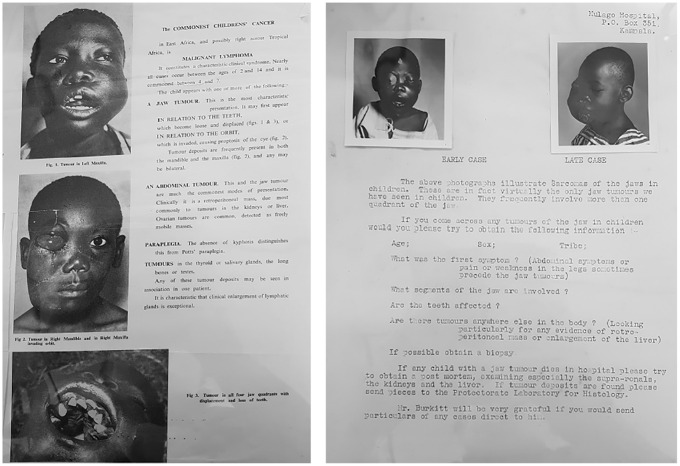
Information leaflet and questionnaire sent by Burkitt for his postal survey. Source: Wellcome Collection (File WTI/DPB/B/3/1).

**Figure 5.**
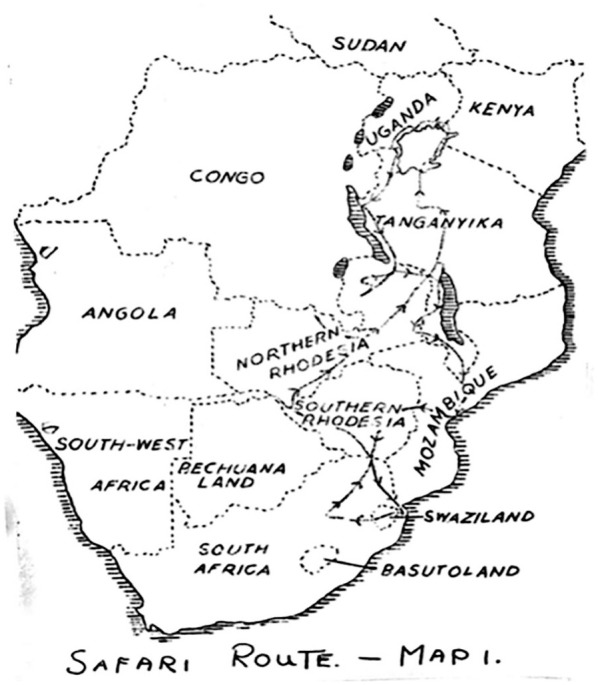
Burkitt’s hand-drawn map of the 1961 safari route. Source: Wellcome Collection (File WTI/DPB/D/1/3).

Burkitt’s safari method was a critical part of the surveillance infrastructure underpinning his lymphoma maps, as it provided the formula for collecting the data on which they were based. Specifically, to collect the data for his maps, Burkitt carried out one postal survey and three safaris in the early 1960s. The postal survey came first. Burkitt designed and printed an ‘illustrated leaflet depicting the characteristic features’ of the tumour along with a ‘questionnaire asking the recipients whether they had observed [it]’, which he sent to medical units across Africa—including ‘British East Africa’, the ‘Portuguese territories of Mozambique and Angola’, ‘the Belgian Congo’, and ‘French West Africa’ (Burkitt, 1961a, p. 512; Burkitt, 1962c, p. 379; Burkitt & O’Conor, 1961, p. 261; see Figure 4). The results of the postal survey seemed to indicate a possible ‘tumour belt’ running across the middle of Africa. To confirm this finding and accurately track the edges of this tumour belt, Burkitt believed it was essential to travel along the path of his map—literally moving his body across the geographical terrain he had charted. This resulted in a 10-week and 16,000-kilometre-long safari from Kampala to Johannesburg and back, which Burkitt did together with two companions: Ted Williams, a British physician who ran a mission hospital in Northwest Uganda, and Cliff Nelson, a Canadian who had worked as a mission doctor and a colonial medical officer in British East Africa (Glemser, 1970). Using an old 1954 Ford Station Wagon, they drove across eight countries following an itinerary mapped by Burkitt, visiting over 50 hospitals and asking doctors whether they had seen the tumour (Burkitt, 1962c; see Figure 5). To further confirm his findings, Burkitt later flew onboard a light aircraft to the Belgian colony of Ruanda-Urundi for a three-day safari, speaking with doctors in hospitals in Bujumbura and Kigali. He then carried out a three-week safari by plane and train across the Congo, Nigeria, and Ghana, visiting hospitals in cities such as Leopoldville, Lagos, and Accra (Burkitt, 1962c).

**Figure 6.**
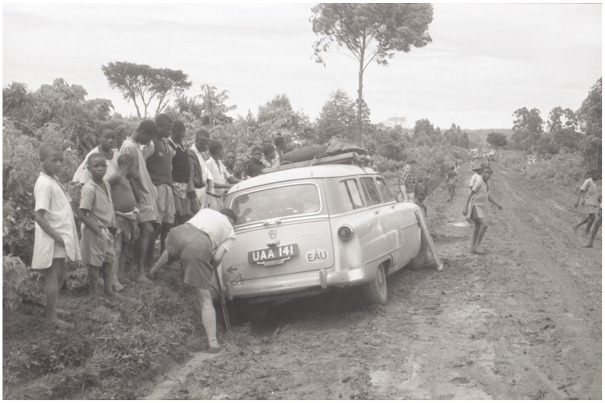
Burkitt’s Ford station wagon marooned in the mud on the return leg, north of Kisumu. Source: Photographic archive of the family of Denis Burkitt.

Significantly, Burkitt’s safari method relied on two often overlooked but crucial technologies—the imperial road and postal networks that covered the colonial territories he visited. Roads—usually ‘simple earthen roads’ made using ‘cheap, manual [African] labour’—and a functioning mail service were key tools of colonial administration and control (Freed, 2003, pp. 203–204). They created a ‘sense of connectedness’ by linking administrative centres with isolated missionary outposts, and enabled colonizers to ‘move around their territory’ and exchange information (Freed, 2003, pp. 203–204). Colonial doctors relied heavily on these networks, from travelling to remote clinics for inspections, to shipping tissue samples for analysis, to advising on difficult cases by post (Crozier, 2007; Greenwood, 2016). As a colonial doctor himself, Burkitt was firmly embedded in these networks and used them to collect the data for his lymphoma maps. He used the postal network not just to send copies of his questionnaire to doctors across Africa, but also to coordinate his tumour safaris, writing ahead to hospitals to inform them of his arrival and the object of his research (Burkitt et al., 1970). The road system was equally critical for Burkitt’s research, with him and his two companions driving thousands of kilometres on robust tar roads or rough dirt tracks linking the government and mission hospitals they visited. Echoing Bowker and Star’s work, the importance of this African imperial road infrastructure, together with the Ford Station Wagon that Burkitt and his companions drove on it, only came to the fore when they broke down. This, for example, was the case when the three men got a flat tire while driving across Tanganyika, forcing them to spend a night in a rest house and delaying their research (Glemser, 1970, p. 76). Or, more seriously, when they had to cancel the last leg of their long safari to Rwanda because the rains had made the roads ‘impassable’, with whole sections ‘washed away’ and their vehicle repeatedly getting ‘stuck in the mud’ (Burkitt, 1961b, p. 72; Williams, n.d., p. 25; see Figure 6).

Another distinctive feature of Burkitt’s surveillance infrastructure was the weight it gave to testimonies of experienced, almost always white, doctors. Burkitt’s main source of information about the presence or absence of the lymphoma in an area was the locally stationed doctor. His postal survey, for example, was addressed directly to doctors, asking them whether they had ‘come across any tumours of the jaw in children’ while working in their hospitals and, if so, to describe the patient’s ‘symptoms’ (see Figure 4). Likewise, in his tumour safaris, Burkitt mostly met and talked with doctors, trying, as he states, ‘to probe into the experience of the local doctors’ and ‘see as many clinicians as possible, the radiologist and, if possible, the chief medical officer’ (Burkitt, 1960, p. 2; Hutt & Burkitt, 1965, p. 719). Given that in Africa biopsy rates were low and hospital records often non-existent, Burkitt argued that ‘the authoritative statement of competent clinicians as to the frequency of a well-defined syndrome [like the jaw tumour] was of great value’ and, emphasized that it ‘must be taken into consideration’ (Burkitt, 1963a, p. 2, 1969, p. 89). This was especially the case with a condition like the lymphoma, whose impact on the children’s faces was visually stark and recognizable. As Burkitt explained, ‘the fact [that] the strikingly characteristic jaw tumour … cannot easily be mistaken for any other condition’ could be used to reliably determine the ‘existence or absence of the syndrome in any area’ and its ‘geographical distribution’ across the continent (Burkitt, 1962b, p. 233, 1962d, p. 1020). Burkitt (1964b, p. 1) especially valued testimonies from ‘doctors with a long service’ and ‘experience’ in a particular area. For example, he argued that the fact that ‘five doctors with experience of up to 24 years of African medicine in the Rhodesias’ had never encountered the lymphoma was strong evidence that it did not exist in that region (Burkitt & O’Conor, 1961, p. 261).

Although Burkitt readily recognized that his safari method, on which his maps rested, was somewhat impressionistic and ‘seldom accurate’, he still believed that it could generate valuable ‘misty images’ that could help ‘convey impressions’ about disease patterns that could be explored in more detail later. Put differently, safaris were not about ‘details or absolute figures’; they could only offer ‘imprecise figures’ and ‘a rough measure of the existence of [a] disease’ (Burkitt, 1963a, p. 2; Burkitt & O’Conor, 1961, p. 260). While ‘dubious’ and ‘useless’ to the ‘statistical purist’, for Burkitt these figures still offered ‘impressions of frequency’, which pointed to where it might be worth engaging in ‘more detailed studies’ (Burkitt, 1963a, pp. 1–2; Hutt & Burkitt, 1965, p. 722).

## Surveillance infrastructures II: Map consciousness

Burkitt also relied on mapping practices as a form of surveillance infrastructure, using them to interpret and make sense of the lymphoma incidence data he had collected during his safaris. Like the safaris, these mapping practices came from colonial medicine and were ubiquitous in medical work across British East Africa at the time, from colonial medical officers running their districts, to entomologists studying the ecologies of infections, to geographical pathologists working on cancer.

Bryan Langlands, a geographer who studied the ecology of tsetse flies at Makerere at the same time Burkitt was there, provides a first-hand account of the role that maps and mapping played in medicine in Kampala and British East Africa in the 1950s and early 1960s. Reflecting on what he was seeing, Langlands (1969, p. 9) noted that ‘the map consciousness’ and ‘enthusiasm for a geographical approach to medical problems’ that existed among doctors in Kampala and British East Africa was ‘unparalleled elsewhere in the world’. He described Makerere Medical School and Mulago Hospital as an ‘impressive geographic research laboratory’ where ‘geographic thinking prevailed in many departments, particularly in pathology, microbiology, preventive medicine, and medicine’ (Langlands, 1969, p. 9). Specifically, he recounted how, in the 1950s, the medical school and hospital established a ‘Committee for Geographic Medicine’ and a ‘map room’ where a ‘collection of atlases and texts of the local geography was brought together, and a supply of base maps was made available’ to medical researchers (Langlands, 1969, p. 10). He further reported that doctors were encouraged to pay attention to ‘disease distributions’ and to ‘set these distributions against the relevant environmental and vector factors’ (Langlands, 1969, p. 13). They were also taught to use cartographic techniques such as ‘cartographic plotting’ and ‘comparative map analysis’ (Langlands, 1969, p. 12).

The ubiquity of cartographic thinking at Makerere and among doctors in British East Africa reflected the central role that mapping played in colonial medicine (Koch, 2017). In the late colonial period, there were two areas of colonial medicine where mapping was particularly important. The first was research into vector-borne diseases, such as yellow fever and malaria, conducted by tropical medicine experts and entomologists across Africa (Cummiskey, 2020; Webel, 2015). Building on notions of disease ecology, these researchers viewed vector-borne diseases as the product of complex relationships between climate, soil, plants, pathogens, animals, and humans, which could be untangled using laboratory techniques and ecological surveys. Maps were critical to these researchers’ work, enabling them to geographically locate and visually inspect the relationships between disease incidence and environmental factors. The second area was the daily work of colonial medical officers (Koch, 2017). These officers always had ‘maps on [their office] walls’ and an ‘interest in the distribution of environmental phenomena in relation to [disease] patterns’; most of them were ‘experts on the geography of their district’, systematically ‘mapping the distributions of rainfall and populations, etc. against which they compare[d] their maps of helminths, tropical ulcers, and such like’ (Langlands, 1969, p. 11). These cartographic practices had long imperial genealogies, dating back to the development of medical geography and medico-topographical surveys during 19th-century European colonial expansion (Arnold, 1993; Moussy, 2015). As tools for understanding and governing the tropical lands of jungles and fevers opened through military conquest, maps quickly became central to medical practice and knowledge-making in the colonies, reinforcing a ‘fascination with physical geography’ and ‘the relationship between climate, race, and health’ (Osborne, 2000, p. 33).

As a colonial medical officer working in British East Africa, Burkitt was intimately familiar with mapping and its usage in colonial medicine. During his first posting in the small town of Lira, Northern Uganda, he spent a lot of time mapping the distribution of diseases in the district for which he was responsible (Cummings, 2022, pp. 92–93). Later, when working as a surgeon at Mulago, he was immersed in and shaped by the fervent enthusiasm for cartographic thinking and cartographic tools that dominated medical circles in Kampala (Langlands, 1969). As part of his medical work, he also interacted with many tropical disease specialists who used maps in their research, such as Alexander Haddow, a yellow fever researcher at the Uganda Virus Research Institute and a friend of Burkitt (1961b). Given these influences and experiences, it is unsurprising that Burkitt drew on cartographic techniques to analyze the incidence data on the lymphoma he had collected through his postal survey and safaris. For him, these techniques were ‘workbenches’ on which he could graphically marshal and experiment with his data to construct and test possible theories about the aetiology of the jaw tumour (Koch, 2011, p. 12).

**Figure 7.**
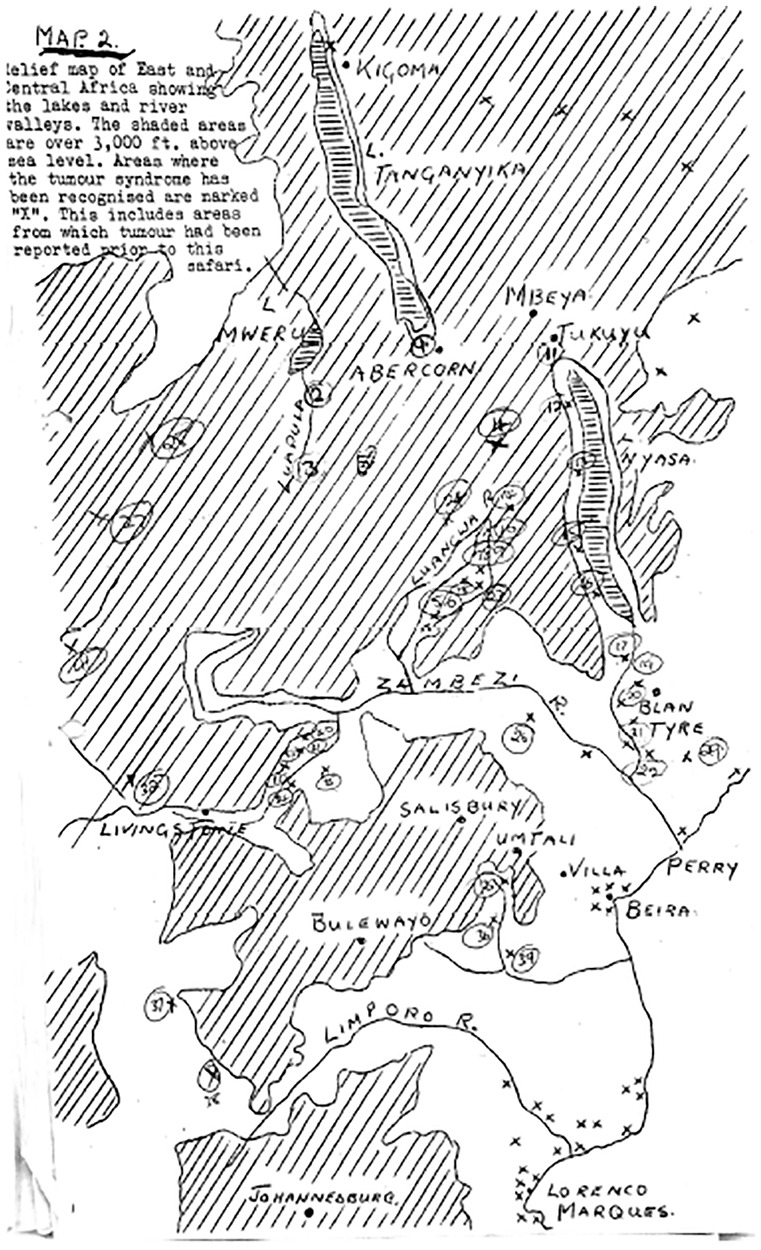
Map of East Africa on which Burkitt has marked with “X” all reported cases of the lymphoma from his postal survey and safaris. Source: Wellcome Collection (File WTI/DPB/D/1/3).

**Figure 8.**
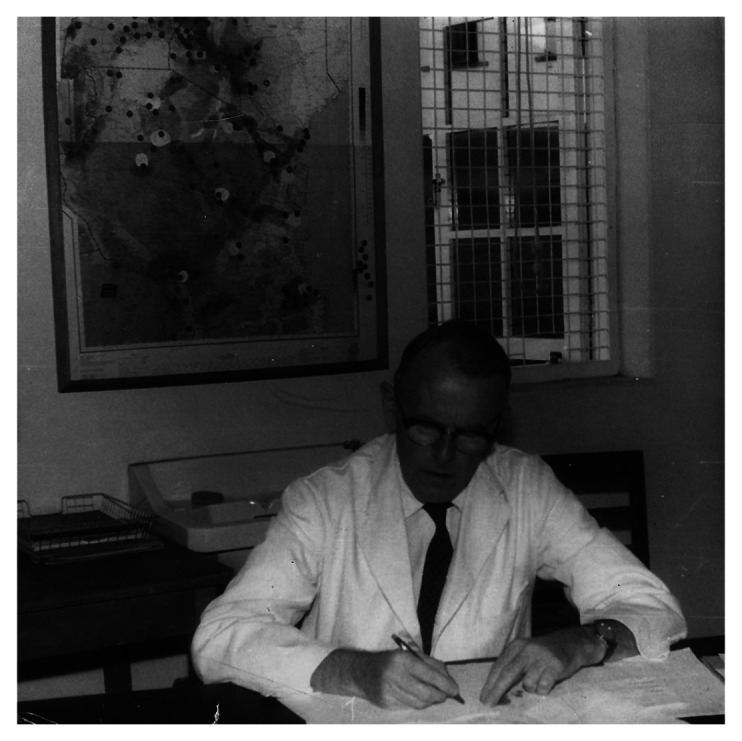
Burkitt in his office at Mulago Hospital in front of a map of East Africa with push pins marking observed cases of the lymphoma. Source: Photographic archive of the family of Denis Burkitt.

To produce his lymphoma maps and support his argument about the lymphoma’s viral origin, Burkitt drew on two cartographic techniques widely used by doctors in British East Africa at the time. The first one was called cartographic or geographical plotting. As Burkitt explained, ‘geographical plotting’, where a researcher marks ‘all cases observed’ of a disease on a map, was ‘the first exercise’ in any ‘study of the geographical distribution of … cancer aimed at relating incidence to environment’ (Burkitt, 1962b, p. 233, 1968, p. 214). This is exactly what he did with the incidence data on the lymphoma, which he had collected through his postal survey and safaris, pushing pins in wall maps or drawing dots on paper maps to mark the locations where cases of the lymphoma had been reported (Langlands, 1969, p. 12; see Figure 7). Burkitt would often hang these maps in his office at Mulago to study them; as a result, his office walls were ‘covered with maps [with pins] showing the distribution of the tumour in Uganda, East Africa, and Africa as a whole’ (Burkitt, 1961c; see Figure 8).

**Figure 9.**
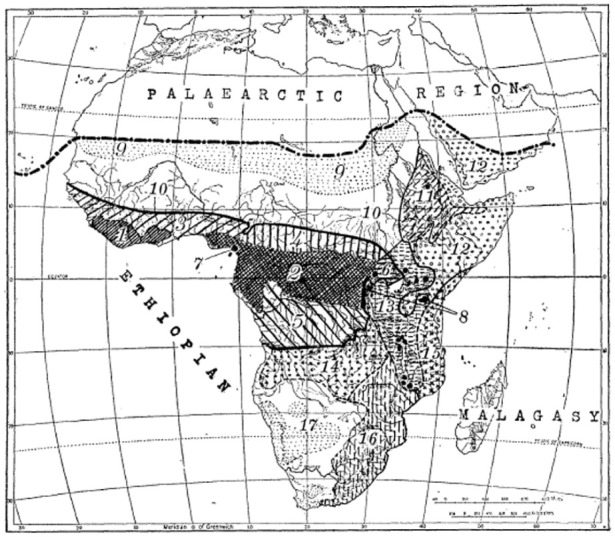
Map of bird distribution in Africa from James Chapin’s 1923 article in *The American Naturalist*.

**Figure 10.**
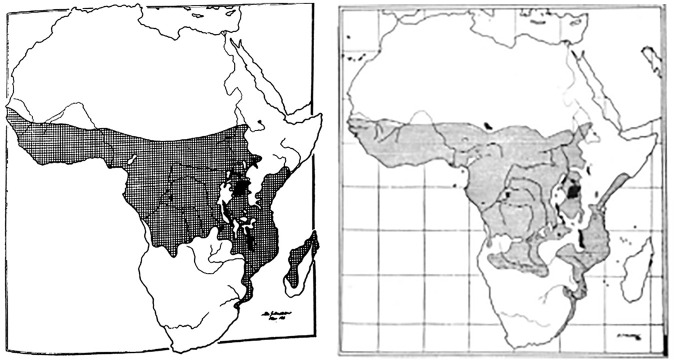
Haddow’s maps of mosquito distribution in Africa, published in Burkitt’s 1961 article in the *East African Medical Journal* (left) and in his 1962 article in the *Annals of the Royal College of Surgeons of England* (right) (Burkitt, 1962g).

The second cartographic technique Burkitt used was comparative map analysis. This involved taking a map showing the distribution of a disease and comparing it with other maps tracing the geographical distribution of possible environmental factors, in the hope of finding aetiological associations (Langlands, 1969). The nature of these other maps depended on the aetiological hypotheses pursued. These included maps of rainfall, altitude, temperature, as well as maps tracking the geographical distribution of soil types, plants, or animals. In his analysis, Burkitt was assisted by his friend Alexander Haddow, an entomologist and yellow fever expert, to whom he showed his lymphoma maps (Cummiskey, 2024). These reminded Haddow (1970, p. 198) of James Chapin’s 1923 maps of bird habitats in Africa, which were ‘familiar to students of insect-borne disease’ like himself and showed how avian habitats correlated closely with ‘vegetation, temperature, and rainfall’. He was especially struck by the ‘close correspondence’ between the lymphoma belt charted by Burkitt and the ‘zoogeographical areas’ mapped by Chapin (Haddow, 1970, p. 198; see Figure 9). For Haddow (1970, p. 198), this correspondence and the fact that Chapin’s zoogeographical zones correlated with the distribution of insects, ‘suggested strongly that an agent transmitted by arthropods, probably mosquitoes, might be involved’ in the genesis of the lymphoma. To make his point, Haddow produced a ‘map [of Africa] illustrating climatic conditions known to favour the growth of certain mosquitoes’, which Burkitt published alongside his own maps (Burkitt, 1961a, p. 514; see Figure 10). The similarity between Haddow’s map and Burkitt’s lymphoma belt added ‘strong support to the theory of virus infection dependent on an arthropod vector’ (Burkitt, 1961a, p. 514). Burkitt’s maps, therefore, emerged from a recursive epistemic process that built on earlier colonial cartographic practices.

## Human infrastructures: Colonial medical communities

Alongside the epistemic and surveillance infrastructures described above, Burkitt’s maps and his hypothesis about tumour viruses were also subtended by a deeper, less visible form of infrastructure—the networks of white, English-speaking doctors and their families spread across British Africa in the final decades of colonial rule. These ‘colonial medical communities’, as Crozier (2007, p. 115) has called them, were composed of a small number of white doctors and their families who worked in mission and government hospitals across the continent, united by shared ideas and lived experiences in Africa during the time of empire.

The shared ideas that held these communities together were multiple. One of them was the conceptualization of race and racial difference. Hospitals across British East Africa were highly segregated: Most senior doctors were white, while subordinate roles such as nurses and medical assistants were typically held by Africans (Iliffe, 1998). These senior doctors were considered part of British Africa’s ‘white, ruling-class minority’, ‘bound by the colour of their skin’ to other ‘colonials, whether settlers, missionaries, or government officials’ (Crozier, 2007, pp. 115, 124). Ideas of race and racism were often justified by theories about empire and its civilizing mission, which portrayed Africans as ‘immature’ and in need of ‘moral and social guidance’ from Europeans (Greenwood, 2016, p. 74). Colonial doctors were also united by their shared imaginaries of Africa as a place of adventure and Christian good works (Vaughan, 1991). These imaginaries had roots in the romantic representations of exploration, travel, and empire found in novels and writings of pioneers and adventurers like David Livingstone and Henry Stanley. In these writings, Africa was pictured as ‘a strange land full of exotic creatures and people’ where ‘the game was bigger [and] the skies wider’ (Crozier, 2007, p. 63). Being a doctor in Africa meant partaking in the ‘imperial life of adventure’, where ‘brave, selfless’ heroes, informed by Christian-cum-humanitarian ethics, ‘endured the hardships of an unhealthy and uncivilized place’ (Crozier, 2007, p. 63).

Colonial medical communities in British Africa were also held together by shared social and professional backgrounds and practices. Doctors and their families usually lived with other English-speaking Europeans in ‘enclaves’ that were ‘physically distinct from the African communities around them’, where they ‘forged friendships and lifelong loyalties’ (Crozier, 2007, p. 118). Life in these enclaves was organized around standard colonial rituals—from church worship and dinner invitations to tennis and sundowners at the country club, to picnics, big game hunts, and celebrations of the monarch’s birthday. The organization of these rituals was often left to women, who were tasked with creating a ‘home-from-home’ and ‘normaliz[ing] the otherwise exotic’ colonial life (Crozier, 2007, p. 101). For doctors—almost all of whom were men trained at universities in London, Edinburgh, or Dublin—life was further organized around professional rituals. These included conversations with colleagues on the wards or during medical safaris, regular scientific meetings at the local branch of the British Medical Association, and an informal culture of medical journal swapping (Crozier, 2007; Greenwood, 2016).

**Figure 11.**
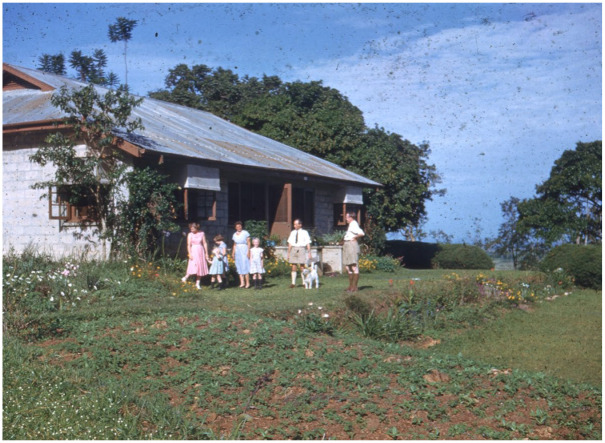
‘A home-from-home’: Denis Burkitt and his family hosting friends at their villa on Mulago Hill, Kampala. Source: Photographic archive of the family of Denis Burkitt.

In many ways, Burkitt was a typical member of the postwar colonial medical community in British East Africa. By the time he published his maps, he had been working for the British Colonial Medical Service in Uganda for 15 years, first as a medical officer in Lira and then as a surgeon at Mulago. As was usual among colonial doctors, he also engaged in research, publishing on an extraordinarily all-encompassing range of subjects, from abdominal surgery to prosthetic legs (Vaughan, 1991). Moreover, like other colonials, Burkitt was infused by the ‘adventurer’ spirit. In his diaries, he wrote that living in Africa ‘was the greatest adventure of my life’ and spoke fondly of his early years in Uganda using a bucket toilet and a hurricane lamp (Burkitt, cited in Cummings, 2022, p. 83). Born in an evangelical family and deeply religious himself, Burkitt also saw his medical work in Africa in strongly Christian terms, thinking that his presence on the continent was the result of ‘God’s leading’ (Burkitt, n.d., p. 2). In Kampala, Burkitt and his family lived in the European part of the town in a large, bucolic villa on Mulago Hill, where they were waited upon by African servants (see Figure 11). Apart from work, Burkitt’s life revolved around Kampala’s Anglican church, where he was active in the Bible study group, and the Uganda Club—a white-only members club for officers of the colonial administration. With his wife, Olive, he also entertained at home, played tennis, picnicked on the shores of Lake Victoria, participated in the pageantry of empire, and went on safaris in game parks (Cummings, 2022).

Burkitt actively drew on his membership of the British East African medical community in his research on the lymphoma, especially to conduct his postal survey and tumour safaris. To begin, his safaris relied on the network of district hospitals and rural clinics run by colonial medical officers and missionary doctors set up from the 19th century onwards. During the long 1961 safari, Burkitt and his two companions visited 56 hospitals—29 operated by Christian missions and the rest, except for those in South Africa and Portuguese East Africa, run by the British colonial administration (Glemser, 1970). Many of the medical professionals they visited were already known to them, and when this was not the case, they often shared similar professional, social, and educational backgrounds. Indeed, during the trip, the three men encountered numerous graduates from Trinity College Dublin, Burkitt’s alma mater, met doctors with mutual acquaintances, and even came across many medical practitioners from Burkitt’s hometown of Enniskillen in Northern Ireland (Cummings, 2022). Reflecting on his safaris, Burkitt (1963a, p. 1) remarked that ‘investigations of this sort can only succeed if … [the investigator] has extensive personal contacts with the medical personnel in the area to be investigated.’ Indeed, he believed that ‘establishing … personal contacts with hospital staff is … of the utmost importance’; ‘colleague to colleague, and where possible, friend to friend must be the relationship sought’ (Burkitt, 1963b, p. 19).

Most doctors with whom Burkitt corresponded or spoke as part of his postal survey and tumour safaris were of European descent—from Britain, France, Germany, or Portugal (Glemser, 1970). Very few non-white people were called upon in the map-making process, and there was no mention of black doctors in Burkitt’s diaries. Indeed, Burkitt seemed dismissive of African doctors, confiding to Himsworth (1963) at the MRC that while white doctors in ‘mission hospitals were extremely helpful in providing information’ about the lymphoma, ‘very little information had … come from African surgeons’ (p. 2). This chimed with ideas of race and racism that dominated the medical community in British East Africa at the time, with the few practising African doctors restricted to subordinate roles and having no research opportunities (Iliffe, 1998). And it was also consistent with Burkitt’s own beliefs about African difference and inferiority. While never stated explicitly, these beliefs were evident in Burkitt’s belittling remarks about African elites—for instance, referring to Milton Obote, Uganda’s first independence leader, as ‘the boy’ who had ‘looked after goats in a remote … village’, or characterizing the ‘African Minister of Health of Urundi’ as a former office ‘clerk’ who ‘did not register a great deal of intelligence’ (Burkitt, 1962e, p. 2). These beliefs are also evident in Burkitt’s conviction that the British Empire had been beneficial for Africans, liberating them from ‘the oppression, cruelty, superstition, fear, and disease which blighted [their] life’ before colonialism (Burkitt, 1962f, p. 2).

Less tangible than the networks of white, male doctors, but no less important, were the colonial adventurer spirit and Christian ethics that underpinned and shaped Burkitt’s tumour safaris. As Burkitt argued, these safaris were not just an ‘interesting and profitable’ research technique, but also an ‘enjoyable’ personal experience offering ‘a glow of real adventure’ (Burkitt, n.d., p. 1; Burkitt et al., 1970, p. 201). For example, during their long safari, Burkitt and his two companions had a thrilling time, driving along deserted roads, admiring the landscapes, spotting wild animals, contending with the African bush, and visiting famous sites like the Kruger National Park and Victoria Falls (Glemser, 1970). In the evenings, they often went for dinner and sundowners and frequently stayed at the opulent homes of missionaries, settlers, and colonial officers (Burkitt, 1961b, pp. 4, 28; Williams, n.d., p. 22). The three men also went to church on as many Sundays as possible and took great pleasure in staying with Christian doctors like Oettlé and their families, often joining in evening prayer and Bible reading (Cummings, 2022). Notably, Burkitt and his companions frequently imagined their safari through the lens of David Livingstone’s African travels. Deeply inspired by the 19th-century doctor-cum-missionary, the three men had taken a copy of Livingstone’s travel diaries with them and read it regularly. They wrote of their joy in following in Livingstone’s footsteps, ‘picturing him walking though the bush day after day’, ‘traversing [a particular] river’, or ‘tramping along in that intense heat’ (Burkitt, 1961b, pp. 23, 25; Williams, n.d., p. 6). They also planned their safari route with stops at places associated with Livingstone, such as Ujiji on Lake Tanganyika, where Stanley met Livingstone, which Burkitt described as ‘an emotional experience’ (Burkitt, 1961b, p. 6).

The rarely noticed infrastructural work performed by medical communities in British Africa in support of Burkitt’s maps became especially evident when he left them behind to travel to Portuguese East Africa and Francophone West Africa for his safaris. The visit to Beira in Mozambique, which Burkitt carried out as part of his long safari, is a good example. In his diary, Burkitt (1961b, p. 26) wrote feeling ‘awfully deserted in a strange country’. Everything seemed different: the ‘extremely modern’ architecture with ‘bizarre colours and totally unorthodox shapes’ (Burkitt, 1961b, pp. 28–29), the ‘terrible’, ‘high-pitched whining of low-capacity autocycles’ (Burkitt, 1961b, p. 28), and the unfamiliar, ‘oily’ food served in ‘rather strong sauce’ (Burkitt, 1961b, pp. 27, 41). The hospital, too, felt different: ‘The sisters were nuns’ (Burkitt, 1961b, p. 28), ‘there were all kinds of specialists’ unfamiliar to him (Williams, n.d., p. 12), and there was ‘no official colour bar’, with ‘Africans and Europeans work[ing] side by side’ (Burkitt, 1961b, p. 38; Williams, n.d., p. 12). One difference was particularly important: language. As Burkitt (1961b, p. 28) recounted, he spent ‘most of the morning’ at the hospital ‘trying to describe [his] tumour to Portuguese doctors’; and it was only when a doctor who had ‘worked in America’ came to ‘help with interpretation’ that he got any information about the lymphoma. This made a method based on testimonies particularly fragile. After driving back into British Rhodesia, Burkitt (1961b, pp. 28–29) seemed relieved: The ‘green country with mists hanging over the hills’ and the ‘well-kept parks and shortly-cut lawns’ made him feel that he had ‘returned to civilization’.

## Decaying infrastructures

Infrastructures are ephemeral, fragile constructions prone to decay. As Gupta (2018, pp. 62, 76) notes, they are ‘always already on the way to becoming ruins’ and will, without ‘continuous work of maintenance’ and ‘investment’, deteriorate and fall apart. The infrastructural assemblage undergirding Burkitt’s tumour maps was no different in that respect. In 1964, Burkitt left Kampala and the colonial medical service and relocated to London to join the MRC Statistical Unit. There, drawing on the same cartographic infrastructures that had supported his lymphoma work, he began investigating oesophageal cancer, mapping its geographical distribution in East Africa to understand the role diet played in its aetiology. This new research involved setting up a network of mission hospitals across East Africa, which posted monthly reports to Burkitt, and conducting regular tumour safaris across the region (Cummings, 2022). However, African independence and shifts in cancer research led to the irredeemable decline of the epistemic, material, and human infrastructures on which Burkitt relied, making it increasingly difficult for him to conduct his work.

Burkitt’s studies in geographical pathology of cancer took place during a time of upheaval on the African continent. A wind of change was sweeping across Africa as countries fought and won battles for their political independence, from Ghana in 1957 to Kenya in 1963 (Cooper, 2002). Burkitt witnessed this period first-hand. For example, during the 1961 safari, he and his companions literally travelled across political temporalities. On the last leg of their journey, the group drove through Tanganyika on the very night of its independence, going to sleep in a British colony and waking up in an independent country (Glemser, 1970). Burkitt felt apprehensive about decolonization, describing in his diaries the disorder, violence, and decline he witnessed across the continent at the time (Cummings, 2022). He particularly worried that decolonization would make it difficult for him to carry out research in Africa, hindering his ability to conduct tumour safaris and disrupting the networks of white doctors he so relied on. Indeed, a major consequence of independence was Africans ‘taking over the institutions of colonial medicine’ from health ministries to hospitals, while the European doctors and colonial officers departed *en masse* (Iliffe, 1998, p. 118). For Burkitt (1962e, p. 3), this European ‘exodus’ represented a serious blow to his research ambitions, as it led to the disintegration of the colonial medical communities that were critical to his work. He felt that the ‘departure [of European] trained medical observers with their fund of experience’ and ‘the information they [had] gleaned over the years’ would make an ‘inquiry of the nature’ that he had conducted on the lymphoma impossible (Burkitt, 1962d, p. 1021). Burkitt also feared that independence would lead him to lose the privileged access to vast, pacified African territories which the empire had granted him as a colonial medical officer, thereby jeopardizing his ability to carry out tumour safaris. Specifically, he believed that the ‘political turmoil’ associated with decolonization would create ‘frustrations’ for the ‘medical research traveller’, from the need for ‘special [travel] permits’ to the cancellation of research trips due to unrest, fighting and ‘civil war’ (Burkitt, 1964b, p. 1; Burkitt et al., 1970, p. 200).

As African independence dismantled the surveillance and human networks behind Burkitt’s cancer research, shifts in cancer science from the 1960s eroded the epistemic foundations of geographical pathology. First, the development of viral oncology, which Burkitt’s lymphoma maps had accelerated, was ‘pushing the field toward specific forms of molecularization’ (Mueller, 2019, p. 524). From Epstein’s identification of virus particles in cultured cells taken from a Ugandan boy with Burkitt’s lymphoma to the development of alpha-fetoprotein blood tests for diagnosing liver cancer, this emerging field used the machinery of molecular biology to illuminate the chemistry of oncogenesis. Second, the rise of descriptive epidemiology, enabled by advances in computational power and statistical techniques, was shifting the field’s interest in cancer aetiology to a concern with managing cancer control programmes (Cochrane & Reubi, 2023). By the early 1970s these new research traditions had rendered geographical pathology largely irrelevant, with funding flows drying up and the UICC Committee on Geographical Pathology disbanding in the late 1960s. Ultimately, these political and epistemic shifts pushed Burkitt and his impressionistic safaris-as-survey methodology to the margins of cancer research. In 1976, Burkitt, who admitted ‘know[ing] virtually nothing about statistics’ and the ‘language of cell chemistry’, had his MRC funding terminated and was asked to vacate his desk at the Statistical Unit (Burkitt, cited in Cummings, 2022, p. 215; see also Burkitt, 1963c).

## Conclusion

In this article, we articulated a new approach to the study of disease maps, using Denis Burkitt’s influential lymphoma charts to illustrate its analytical force. Rather than being concerned with the world-making power of maps, our approach focuses on what we have termed ‘cartographic infrastructures’—the rationalities, materialities, and socialities that make the production of maps and the stories they tell about the world possible. In these concluding remarks, we want to restate and expand on some of the analytical insights that an infrastructural approach can offer to social scientists and historians studying maps and mapping efforts in global health and biomedicine.

To take an infrastructural approach is to illuminate the ‘behind-the-scenes, boring, background’ arrangements, objects, and people that do ‘the real work of knowledge production’ (Bowker & Star, 1996, p. 234). Applied to cartographies of disease, this approach allows one to look behind maps and bring to the fore the often-invisible infrastructural assemblages that undergird their formulation and existence. As we showed in our analysis of Burkitt’s tumour maps, these assemblages are rich and multifaceted. They comprise epistemic infrastructures, such as the aetiological theories, scientific forums, research facilities, and funding flows that were associated with geographical pathology and gave Burkitt the intellectual, material, and financial tools to conduct his lymphoma research. They also encompass surveillance infrastructures, such as the postal surveys, tumour safaris, and geographical plotting techniques that Burkitt used to collect and comprehend the data found in his maps. As we outlined, these methodologies themselves relied on material infrastructures—ranging from the paper questionnaires used in the postal surveys, to the imperial road networks that enabled the tumour safaris, to the pins and wall maps employed to plot the lymphoma’s distribution across Africa. Lastly, these assemblages also include human infrastructures, like the networks of white, mostly English-speaking colonial doctors with their shared imaginaries of adventure and exploration on which Burkitt relied to know where the lymphoma occurred on the continent. The approach we articulated in this article enables us to draw attention to all these often invisible infrastructures and show the essential role they play in cartographic knowledge-making.

An infrastructural approach also enables us to shine a light on the moral ambiguities of some of the infrastructures undergirding disease maps. These infrastructures are often embedded in unequal power dynamics and legacies of injustice and exploitation. The research tradition of geographical pathology that scaffolded Burkitt’s maps was rooted in Enlightenment and imperial imaginaries of Africa as other—primitive, unsophisticated, and always behind Euro-America. Geographical pathology was also a deeply unequal and extractive scientific project, in which European and some American doctors and funding agencies used Africa and Africans as a laboratory for understanding and preventing cancer in imperial metropoles. The safari-as-survey methodology reproduced a colonial practice of surveillance, penetration, and control on a grand cartographic scale. Notions of race and racial segregation, which were so pervasive in the colonial medical communities in which Burkitt lived and worked, shaped his decision to mostly rely on the testimonies of white doctors to map the geographical distribution of the lymphoma.

Finally, a concern with infrastructures is an opportunity to draw attention to the fact that, for all their world-making powers, maps and the knowledge they carry are inherently fragile. This cartographic fragility is multiple. First, the production of maps can be highly contingent. For instance, Burkitt’s incidental encounter and discussion with George Oettlé in Kampala in 1957 was key in persuading him to embark on his tumour safari. Similarly, Burkitt’s fortuitous friendship with Haddow, with his extensive knowledge of past colonial maps of African geography, climate, and fauna, was instrumental to Burkitt’s discoveries. Second, maps are fragile because the infrastructures that support them can breakdown at any moment—such as when the roads Burkitt travelled during his long safari were washed away by the rains, or when the Portuguese colonial doctors in Beira did not speak English, making it impossible to ascertain the lymphoma’s presence until a translator was found. Third, maps are fragile because the infrastructures supporting them are ephemeral. The infrastructural assemblage underpinning Burkitt’s maps, for example, lasted only 15 years before disintegrating in the wake of African independence, molecular biology, and descriptive epidemiology.

A focus on cartographic infrastructures invites social scientists and historians studying contemporary global health maps to unearth the forgotten scientific, material, and social worlds that enabled their production, thereby troubling the self-evident nature of these maps and revealing them as fraught and fragile scientific objects.
